# Genome-Wide Association Mapping and Genomic Selection for Alfalfa (*Medicago sativa*) Forage Quality Traits

**DOI:** 10.1371/journal.pone.0169234

**Published:** 2017-01-09

**Authors:** Elisa Biazzi, Nelson Nazzicari, Luciano Pecetti, E. Charles Brummer, Alberto Palmonari, Aldo Tava, Paolo Annicchiarico

**Affiliations:** 1 Council for Agricultural Research and Economics—Research Centre for Fodder Crops and Dairy Productions (CREA-FLC), Lodi, Italy; 2 Plant Breeding Center, Department of Plant Sciences, University of California, Davis, CA, United States of America; 3 Department of Veterinary Medicine, University of Bologna, Bologna, Italy; Nanjing Forestry University, CHINA

## Abstract

Genetic progress for forage quality has been poor in alfalfa (*Medicago sativa* L.), the most-grown forage legume worldwide. This study aimed at exploring opportunities for marker-assisted selection (MAS) and genomic selection of forage quality traits based on breeding values of parent plants. Some 154 genotypes from a broadly-based reference population were genotyped by genotyping-by-sequencing (GBS), and phenotyped for leaf-to-stem ratio, leaf and stem contents of protein, neutral detergent fiber (NDF) and acid detergent lignin (ADL), and leaf and stem NDF digestibility after 24 hours (NDFD), of their dense-planted half-sib progenies in three growing conditions (summer harvest, full irrigation; summer harvest, suspended irrigation; autumn harvest). Trait-marker analyses were performed on progeny values averaged over conditions, owing to modest germplasm × condition interaction. Genomic selection exploited 11,450 polymorphic SNP markers, whereas a subset of 8,494 *M*. *truncatula*-aligned markers were used for a genome-wide association study (GWAS). GWAS confirmed the polygenic control of quality traits and, in agreement with phenotypic correlations, indicated substantially different genetic control of a given trait in stems and leaves. It detected several SNPs in different annotated genes that were highly linked to stem protein content. Also, it identified a small genomic region on chromosome 8 with high concentration of annotated genes associated with leaf ADL, including one gene probably involved in the lignin pathway. Three genomic selection models, i.e., Ridge-regression BLUP, Bayes B and Bayesian Lasso, displayed similar prediction accuracy, whereas SVR-lin was less accurate. Accuracy values were moderate (0.3–0.4) for stem NDFD and leaf protein content, modest for leaf ADL and NDFD, and low to very low for the other traits. Along with previous results for the same germplasm set, this study indicates that GBS data can be exploited to improve both quality traits (by genomic selection or MAS) and forage yield.

## Introduction

Alfalfa (*Medicago sativa* L. subsp. *sativa*) is the most-grown perennial forage legume globally [1], owing to its high yield, stress tolerance and forage quality and the positive effects on soil fertility of its cultivation [2]. It is widely adopted in feeding of dairy cows, where, however, poor genetic progress for forage digestibility and forage intake traits limits its full potential of utilization [1]. The nutritive value of forage can be assessed by several key parameters, including the protein concentration, the total fiber concentration and the types of fiber present, the digestibility of the forage, and the leaf-to-stem ratio. In particular, the concentration of neutral detergent fiber (NDF) in the forage is highly negatively correlated with forage intake by animals. Acid detergent fiber (ADF) and acid detergent lignin (ADL) concentrations are negatively associated with forage digestibility [3]. In addition, the digestibility of the NDF fraction of forage is a parameter of increasing interest for assessing the efficiency of forage utilization by animals [4]. Higher forage protein content is desirable to reduce (or eliminate) protein supplementation by costly off-farm feeds. Forage quality is associated positively with the leaf-to-stem ratio (owing to the greater quality of leaves relative to stems) and negatively with the degree of stem lignification, both traits becoming less favourable for forage quality with progression of the reproductive stage [1]. Cultivar, maturity stage and harvest time all have an important effect on the main forage quality parameters [5–8]. Forage quality can also be affected by abiotic stresses, especially drought, through direct and indirect effects on plant morphology and physiology [9–11]. Various studies have revealed the presence of variation for forage quality traits, particularly within cultivars [12, 13]. Thoroughly exploiting this variation requires the evaluation of large numbers of genotypes for forage quality parameters across various growing conditions [14], which is both costly and time intensive. Therefore, the development of selection procedures based on molecular marker information could radically streamline and accelerate forage quality improvement.

Recent advances in next generation sequencing technologies have expedited the discovery of SNP (Single Nucleotide Polymorphism) markers, which are being developed into markers at the genome-wide level in a cost-effective manner. These markers facilitate genome-wide association studies (GWAS) and fine mapping of quantitative trait loci (QTL) aimed to marker-assisted selection (MAS), and genomic selection [15]. Genotyping-by-Sequencing (GBS) uses restriction enzymes to reduce genome complexity followed by next generation sequencing to discover and genotype thousands of SNPs in a single step on many individuals [16]. Because the alfalfa genome has not been sequenced and assembled yet, the related model plant *Medicago truncatula* Gaertn. is useful to identify genomic regions of practical value in alfalfa and other legume species [17] (*Medicago truncatula* genome version *Mt4*.*0v1;* https://phytozome.jgi.doe.gov/pz/portal.html#!info?alias=Org_Mtruncatula, http://www.jcvi.org/medicago/) [18, 19].

GBS and other genomic investigations allowed the identification of QTL influencing various agronomic traits in alfalfa, such as biomass yield [20–22], aluminium tolerance [23], drought tolerance [24], salt tolerance [25], freezing tolerance [26] and biomass yield under drought stress [27]. However, there is a paucity of studies regarding alfalfa forage quality traits. Li et al. [28] identified some markers related to biomass yield and concentration of stem fiber fractions using about 300 simple-sequence repeat markers (i.e., an amount largely insufficient to saturate the genome). Some genomic regions were associated with forage quality traits of diploid *M*. *sativa* (subspp. *falcata*, *caerulea* and *hemicycla*) [29] and *M*. *truncatula* [30] in GWAS experiments.

For quantitative traits, the genome-enabled selection of genotypes based on their breeding value, i.e., their value when used as a parent in synthetic varieties (as determined by additive genetic variation) has particular practical relevance for alfalfa and other open-pollinated forage legumes [1]. We have recently shown that genomic selection for breeding value is a promising avenue for improving alfalfa forage yield [21].

We hypothesize that we can associate alfalfa forage quality traits with several specific GBS-generated SNP markers that could be exploited for MAS, and that we can develop genomic selection models that predict alfalfa genotype breeding values for forage quality traits well enough to use in a breeding program. The objective of this experiment was to test our hypotheses in a broadly-based reference population of alfalfa developed from cultivars well-adapted to Mediterranean-climate environments and phenotyped across different growing conditions.

## Materials and Methods

### Plant material and phenotyping

To develop the population used in this experiment, we first developed a strain cross of three cultivars including the old Sardinian cultivar Mamuntanas (fall dormancy 7, on the standard NAAIC scale from 1 = minimum to 11 = maximum) [31], the Moroccan landrace Erfoud 1 (fall dormancy 9) and the Australian cultivar SARDI 10 (fall dormancy 10) to freely intercross using bumble bees (*Bombus terrestris* L.) inside an insect-proof cage [21]. We subsequently intercrossed random plants from the strain cross to increase recombination among the initial populations. From this second-generation population, we used 154 plants in this experiment for genotyping. While being well-adapted to Mediterranean-climate environments, the original cultivars displayed variation for adaptive traits and adaptation pattern [32]. Phenotyping was carried out on the half-sib progenies of the 154 genotypes to assess parental breeding values [1], using seed obtained by free intercrossing by bumble bees under insect-proof cages of the replicated cloned parents. The half-sib progenies were field grown under a rainout shelter equipped with low-pressure sprinklers during 2012 in Lodi, northern Italy (45°19′ N, 9°30′ E, 81 m elevation). No specific permissions were required for this field activity, as it was carried out in the own experimental field of CREA-FLC in Lodi. The study did not involve endangered or protected species. Seeds of all progenies were sown in polystyrene plug-trays in mid-February. Seedlings were field transplanted on April 16 in plots of 0.216 m^2^ (30 cm × 72 cm) that included 4 rows of 9 plants spaced 7.5 cm and 8.0 cm between and within rows, respectively. Eight edge plants were used as borders.

Quality traits were assessed under three growing conditions. One (C1) was represented by a fully-irrigated crop harvested in summer (July 13). For C1 irrigation was applied every 10 days from April 17 for a total of 355 mm. In the second condition (C2), irrigation was suspended for 21 days between June 22 and the harvest day (July 12). The total irrigation received by C2 was 280 mm. C1 and C2 were grown as nearby experiments in the same field, each designed as an alpha lattice with three replications. The third condition (C3) represented an autumn harvest (October 16) of the same plots that were harvested in the C2 experiment. Those plots were fully irrigated starting from September 12, providing a total of 180 mm irrigation until harvest date.

Plots were mown when about 75% of plants had at least one open flower (early flowering). Those within condition were mown simultaneously, given the very limited range of within-condition phenological differences among progenies (3 days at most). Cutting height was 5 cm above the ground level. Biomass yield and leaf-to-stem ratio were recorded after oven drying at 60°C. Leaf blades and petioles were retained as ‘leaves’, while the remaining aerial parts were classified as ‘stems’, in agreement with previous studies [12].

### Fiber content and *in vitro* digestibility

Leaf and stem samples of the progenies in each condition (amounting to 154 progeny × 3 replications × 3 conditions = 1386 samples for each plant component) were analyzed by Near Infrared Reflectance Spectroscopy (NIRS). Spectra were collected by a scanning monochromator (FOSSNIR Systems 6500, Silver Spring, MD, USA) in the spectral range of 400–2500 nm.

A random subset of samples close to 10% (134 leaf and 134 stem samples) was used for chemical determinations of crude protein, NDF, ADF and ADL concentrations, and *in vitro* NDF digestibility after 24 hours (NDFD). Chemical analyses were carried out separately on leaf and stem samples. Plant materials, after oven drying at 60°C, were ground in a Cyclotec 1093 sampling mill (Foss Tecator AB, Höganäs, Sweden) through a 1 mm screen. Chemical analysis and *in vitro* fiber digestibility were carried out as described in previous reports [8, 33]. Crude protein, NDF, ADF, and ADL were analyzed as described in [8], with the addition of microfiber glass filters (1.5 μm) (Whatman Limited) to each crucible, as suggested by [34]. NDFD was assessed using Tilley and Terry’s technique [35] as modified in [36].

A NIRS equation calibrated to predict protein and fiber contents was implemented using a subset of 200 chemically-analyzed test samples (100 leaf and 100 stem samples) plus 165 additional chemically-analyzed leaf and stem samples obtained from other alfalfa experiments. We tested the equation predicting ability by a cross-validation, using chemical data of 68 test samples not used for calibration. For NDFD (where no previous additional data were available), the prediction equation was established on the basis of a subset of 241 chemically-analyzed test leaf and stem samples, using 27 independent test samples for cross-validation. The coefficient of determination (*R*^*2*^) of cross-validations for the prediction models was 0.99 for NDF, 0.99 for ADF, 0.94 for ADL, 0.99 for crude protein, and 0.80 for NDFD. All spectra and reference data were recorded and managed with the WINISI software Version 1.5 (Infrasoft International, Port Matilda, PA, USA).

### Statistical analysis

Plot data of leaf-to-stem ratio, protein and fiber concentrations, and NDFD were subjected to an analysis of variance (ANOVA), testing the variation among progenies (P), among conditions (C), and P×C interactions. Genetic coefficients of variation were computed for each environment as:

$$CV_{g}=\frac{\sqrt{{\sigma^{2}}_{g}}}{\overset{´}{X}}$$

where *σ*^2^_*g*_ is the variance component of genotype and $\overset{´}{X}$ is the trait mean.

Broad-sense heritability ($h_{B}^{2}$) on a progeny mean basis was estimated across conditions as:

$$h_{B}^{2}=\frac{{\sigma^{2}}_{g}}{\left({\sigma^{2}}_{g}+\frac{{\sigma^{2}}_{ge}}{c}+\frac{\sigma^{2}}{ck}\right)}$$

where *c* is the number of conditions, *k* is the number of replications within conditions, and *σ*^*2*^_*g*_, *σ*^*2*^_*ge*_ and *σ*^*2*^ are the estimated variance components for progeny, progeny × condition interaction and experiment error, respectively.

Genetic coefficient of correlation (*r*_*g*_) for progeny response across a pair of conditions *i*, *j* was estimated for each pair of conditions (C1 *vs* C2; C2 *vs* C3; C1 *vs* C3) as [37]:

$$r_{g}=\frac{r_{p}}{\sqrt{{h_{B}^{2}}_{Ci}}+\sqrt{{h_{B}^{2}}_{Cj}}}$$

where *r*_*p*_ is the phenotypic coefficient of correlation, and ${h_{B}^{2}}_{Ci}$ and ${h_{B}^{2}}_{Cj}$ is the broad-sense heritability of the relevant pair of conditions.

Phenotypic correlation coefficients for progeny values averaged over conditions were estimated between quality traits within leaf and stem samples, as well as between leaf and stem values of each quality trait.

Phenotypic trait values for genomic selection and GWAS studies were adjusted using BLUP (best linear unbiased predictors) computed from half-sib progeny mean values, as described in [38]. Statistical analyses were carried out using the softwares SAS and PBTools (PBTools, version 1.4., International Rice Research Institute, Los Baños, The Philippines; http://bbi.irri.org/products).

### DNA isolation, library preparation and sequencing

DNA was isolated from fresh leaf tissues by the Wizard® Genomic DNA Purification Kit (Promega, A1125), and quantified with a Quant-iT PicoGreen dsDNA assay kit (Life Technologies, P7589). The DNA library was constructed using the protocol from [16] with modifications as described in [21], and was sequenced in one lane of Illumina HiSeq 2000 platform at the Genomic Sequencing and Analysis Facility at the University of Texas at Austin, TX, USA.

### SNP calling, data filtering, and missing data imputation

We used the UNEAK pipeline [39] for SNP discovery and genotype calling. The raw reads (100 bp, single end read) obtained from the sequencer were first quality-filtered and de-multiplexed. All reads beginning with the expected barcodes and cut site remnant were trimmed to 64 bp. Identical reads were grouped into one tag.

A further quality filter, implemented through *ad hoc* Python scripts, removed heterozygous loci with less than four aligned reads, and homozygous loci with less than 11 reads. This way, the probability to falsely call an AAAa heterozygote as homozygote was reduced to 4.22%, as detailed in [40]. A similar filtering was performed in [41] using less restrictive thresholds. For each sample, all loci not reaching the required reading depth were considered missing. Details on SNP calling and quality filtering for these genotypes can be found in [21].

The three possible heterozygotes for this autotetraploid species (i.e., Aaaa, AAaa, and AAAa) were marked as diploid heterozygote (i.e. Aa), while the two tetraploid homozygotes (i.e. AAAA or aaaa) were marked as diploid homozygotes (i.e., AA or aa), according to [42].

The resulting data set was further filtered removing all markers having more than 30% missing data across genotypes (i.e., keeping markers with a call rate of at least 70%). The remaining missing data were imputed using KNNI imputation (K = 4) with the R package Scrime [43] as advised by [40], following the procedure detailed in [21].

The SNP data are available in the NCBI’s Sequence Read Archive (SRA) repository at the address: http://www.ncbi.nlm.nih.gov/sra/SRX1421601 in connection with the study by [21].

### Alignment to *M*. *truncatula*, GWAS and marker-trait association analyses

The Bowtie2 tool [44] was used to query the consensus sequence of each tag pair containing a SNP against the *M*. *truncatula* reference genome (*Mt4*.*0v1*) [18, 19] using the verysensitivelocal preset. SNP not aligning were placed in a fictitious chromosome 9 for visualization purposes.

An earlier study highlighted the absence of sub-population genetic structure for this reference population [21]. A GWAS was conducted based on the mixed model in formula:

y=μ+G×u+ϵ

where *y* is the vector of observed phenotypes, *μ* is the mean of *y*, *G* is the genotype matrix (e.g., {0,1,2} for biallelic SNPs), *u* ~ N (0, I*σ*^2^_*u*_) is the vector of marker effects, and *ϵ* is the vector of residuals. Resulting marker effect were adjusted for inflation as described in [45] using the R package GenABEL [46].

False discovery rate in GWAS may be controlled by Bonferroni’s multiple testing correction method, which proved to be too conservative in practice [47], or by the FDR method, whose correction for over-stringency based on local variation of linkage disequilibrium along the chromosome is extremely difficult [17]. We preferred to use a conservative *P* level for Type I error rates, namely *P* < 0.001, corresponding to an association score (–Log_10_(*P*-value)) ≥ 3.0, as suggested in earlier studies [29, 48]. *M*. *truncatula*-aligned SNPs that were significantly associated with one or more forage quality traits were mapped on *Mt4*.*0v1* using the Jbrowse tool in the plant comparative genomics portal Phytozome (https://phytozome.jgi.doe.gov/pz/portal.html#!info?alias=Org_Mtruncatula) [49] to identify candidate genes. The graphical representation of alfalfa markers mapped on chromosomes was generated using MapChart 2.3 (https://www.wageningenur.nl/en/show/Mapchart-2.30.htm) [50].

### Regression models

We implemented different statistical models for genomic selection [51, 52]. We tested Ridge-regression BLUP, three Bayesian models, and one Support Vector Regression model that proved valuable in a previous study [21]. The accuracy of predictions was assessed by Pearson’s correlation between predicted and observed phenotypes in a ten-fold cross-validation scheme. The whole procedure was repeated 500 times, averaging the resulting accuracies, to ensure numerical stability.

Ridge-regression BLUP (rrBLUP) assumes a linear mixed additive model where each marker is assigned an effect as a solution of the equation (for equation details see previous section):

y=μ+G×u+ϵ

Solving with the standard rrBLUP method, the solution was:

$$\hat{u}=G^{\prime }(GG^{\prime }+\lambda l)-1(y-\mu )$$

where $\lambda =\frac{{\sigma^{2}}_{e}}{{\sigma^{2}}_{u}}$ is the ridge parameter, representing the ratio between residual and markers variance [53]. Given the vector of effects, it is then possible to predict phenotypes and estimate genetic breeding values. Ridge-regression BLUP analysis was performed using the R software package rrBLUP [54], estimating *λ* in a restricted maximum likelihood scheme implemented by a spectral decomposition algorithm, and solving the resulting linear model.

Bayesian-based models assign prior densities to marker effects inducing different types of shrinkage. The solution was obtained by sampling from the resulting posterior density through a Gibbs sampling approach, as described by [55, 56]. We examined the phenotype prediction performances of three Bayesian prediction models, namely: (i) Bayes A [57]; (ii) Bayes B [58]; and (iii) Bayesian Lasso [59]. Bayesian models were investigated by the R software package BGLR [60] using the following parameters: number of iterations = 3,000; burn-in = 500; thinning = 5.

Support Vector Regression models are based on the computation of a linear regression function in a high dimensional feature space where the input data were mapped via a kernel function [61]. We considered a linear kernel (SVR-lin) and used the *ϵ*-insensitive regression present in the R software package e1071 [62], which ignores residuals smaller in absolute value than some constant (*ϵ*) and assigns a linear loss function for larger residuals. SVR-lin hyper-parameters were tuned using a grid search on a subset (10% of total data) to find the best configuration. Considered values were *C* ∈ {2^1^ … 2^6^} for the cost parameter, and *ϵ* ∈ {0,0.1, … 0.9} for the sensitive parameter.

## Results

### Phenotypic variation for forage quality traits

Significant differences (*P* < 0.05) among conditions were observed for all traits in the ANOVA, except for ADL in leaves. On average, the autumn growing condition (C3) exhibited definitely better forage quality than two conditions harvested in summer (C1 and C2), owing to markedly higher leaf-to-stem ratio and to more favourable stem characteristics such as higher protein and NDFD and lower NDF and ADL concentration (Table 1). Fully-irrigated (C1) and irrigation-suspended (C2) summer growing conditions showed no difference for forage quality traits except for leaf protein concentration and leaf NDFD, which were lower in C2 (Table 1). Actually, the difference in drought stress level between C1 and C2 was not large, as indicated by the only moderate difference in condition DM yield, namely 2.61 t/ha for C1 *vs* 2.40 t/ha for C2 (while the autumn harvest C3 yielded definitely less, namely, 1.44 t/ha). As expected, leaves displayed lower fiber contents and higher NDFD than stems in all conditions (Table 1).

**Table 1:** Mean values of forage quality traits recorded on dry leaves and stems of 154 alfalfa half-sib progenies in three growing conditions (C1, C2, C3).

Condition^a^	Leaf-to-stem ratio^b^	NDF (% DM) ^b^^,^^c^		ADL (% DM) ^b^^,^^d^		Crude protein (% DM) ^b^		NDFD (% NDF)^b^^,^^e^	
		Leaf	Stem	Leaf	Stem	Leaf	Stem	Leaf	Stem
C1	1.09b	24.67a	66.70a	3.92	13.81a	31.20a	10.71b	52.18a	28.77b
C2	1.03b	24.38a	62.75a	3.74	12.88a	28.84b	10.86b	50.26b	28.45b
C3	1.43a	21.89b	48.55b	4.10	9.41b	31.74a	17.87a	49.67b	44.98a

^a^C1, summer harvest (July 13), full irrigation; C2, summer harvest (July 13), 21 days irrigation suspension prior to harvest; C3 autumn harvest (October 16).

^b^Column mean values followed by different letters were different at *P* < 0.05 according to Duncan’s multiple range test.

^c^NDF, neutral detergent fiber (DM, dry matter).

^d^ADL, acid detergent lignin.

^e^NDFD, *in vitro* NDF digestibility at 24h.

We do not report results for ADF because they paralleled closely those for NDF, with a correlation between ADF and NDF progeny values over environments of *r* = 0.93 for stem values and *r* = 0.84 for leaf values.

Progenies varied consistently in each condition at *P* < 0.01 for leaf-to-stem ratio, leaf protein content, leaf and stem NDFD, and stem NDF and ADL, whereas non-significant variation (*P* > 0.05) occurred for stem protein content in C1 (Table 2). Leaf-to-stem ratio, stem ADL and stem NDFD tended to display relatively greater variation than the other traits according to genetic coefficient of variation (*CV*_*g*_) values and ANOVA *P* values in each condition (Table 2).

**Table 2:** Range of progeny mean values, genetic coefficient of variation (*CV*_*g*_) in three growing conditions (C1, C2, C3), *F* test results for overall progeny × condition (P×C) interaction, genetic correlation for progeny responses across pairs of conditions, and broad-sense heritability on a mean progeny basis over conditions (*h^2^_B_*), for forage quality traits recorded on dry leaves and stems of 154 alfalfa half-sib progenies.

Traits^a^	Range	*CV*_*g*_ (%)^b^			P×C^b^	*r*_*g*_ ^b^			*h*^*2*^_*B*_
		C1^c^	C2^d^	C3^e^		C1 *vs* C2	C2 *vs* C3	C1 *vs* C3	
Leaf-to-stem ratio	1.11–1.26	5.02***	4.91***	6.37***	NS	1.00**	0.86**	0.75**	0.55
Leaf NDF	22.9–24.3	2.13*	2.63***	3.51***	NS	1.00**	0.87**	0.60**	0.52
Leaf ADL	3.75–4.16	3.95*	6.07**	4.57*	NS	0.92**	0.58*	0.92**	0.40
Leaf CP	29.9–31.2	1.31***	1.94**	1.83***	**	0.80**	0.52*	0.67**	0.50
Leaf NDFD	49.5–51.5	1.19**	2.27***	1.89***	*	0.50*	0.13NS	0.59*	0.22
Stem NDF	57.1–60.2	1.48**	1.86**	2.39***	NS	0.54*	0.88**	0.52*	0.37
Stem ADL	11.7–12.3	2.60**	2.70**	3.04***	NS	0.79**	1.00**	0.60**	0.43
Stem CP	12.9–14.2	1.59NS	3.97**	2.57***	NS	1.00*	0.77**	1.00**	0.46
Stem NDFD	32.5–35.2	4.39***	4.39***	1.85***	NS	0.73**	0.72**	1.00**	0.57

^a^NDF, neutral detergent fiber; ADL, acid detergent lignin; CP, crude protein; NDFD, *in vitro* NDF digestibility at 24h.

^b^ *, *P* < 0.05; **, *P* < 0.01; *** *P* < 0.001; NS, not significant.

^c^C1, summer harvest (July 13), full irrigation.

^d^C2, summer harvest (July 13), 21 days irrigation suspension prior to harvest.

^e^C3 autumn harvest (October 16).

Half-sib progeny × condition interaction was significant only for leaf protein content (*P* < 0.01) and leaf NDFD (*P* < 0.05) (Table 2). Genetic correlation coefficients for progeny response across pairs of conditions (measuring the extent of consistent progeny response across conditions) were relatively high (*r*_*g*_ ≥ 0.50; *P* < 0.05) for all traits except leaf NDFD between C2 and C3 (*r*_*g*_ = 0.13; Table 2). Due to the lack of conspicuous progeny × condition interaction for most traits and the aim of drawing sound conclusions on the potential of genomic selection for quality traits across a set of realistic growing conditions, we performed the subsequent genomic analyses based on progeny response values averaged over the three conditions. Even when considering leaf protein, the character with largest P×C interaction, analyses across conditions were justified by the fairly high pairwise genetic correlation coefficients. Broad-sense heritability values over the three conditions were moderate for all traits except leaf NDFD (*h*^*2*^_*B*_ = 0.22; Table 2). The practical interest for crop improvement of this trait was lower than for stem NDFD, which had much lower values than leaf NDFD in all conditions (Table 1).

Various correlations (*P* < 0.01) emerged between forage quality traits, some of which indicated fairly strict trait interrelationships. NDF and ADL contents were correlated in stem (*r* = 0.87) and leaf (*r* = 0.52) samples, and were negatively associated with NDFD in stems or leaves (*r* ≤ –0.52). Protein content was closely related negatively with NDF (*r* = –0.67) and ADL (*r* = –0.61) contents in stems, and positively correlated with NDFD in stems (*r* = 0.60) and leaves (*r* = 0.57). Correlations between leaf and stem values were modest for all traits, ranging from *r* = 0.18 for ADL content to *r* = 0.35 for NDFD.

### GBS data and alignment to *M*. *truncatula*

GBS produced a total of 445,125,819 reads, for an average of 2,890,427 reads per sample. UNEAK identified a total of 97,508 loci. After filtering on read depth and missing rate, a total of 11,450 polymorphic markers were retained for analysis, of which 8,494 (74.2%) aligned to the *M*. *truncatula* genome. The remaining 2,956 (25.8%) not-aligning markers were placed in the fictitious chromosome 9 for visualization purposes.

### GWAS and marker trait association analyses

**Fig 1:**
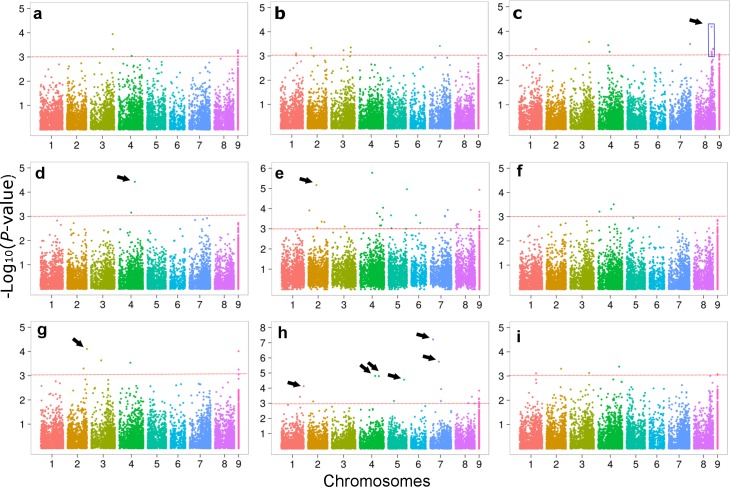
Manhattan plots for genome-wide association of alfalfa forage quality traits with SNP markers. Traits are: (a), leaf-to-stem ratio; (b), leaf neutral detergent fiber; (c), leaf acid detergent lignin; (d), leaf crude protein; (e) leaf *in vitro* NDF digestibility at 24 hours; (f), stem neutral detergent fiber; (g), stem acid detergent lignin; (h), stem crude protein; (i) stem *in vitro* NDF digestibility at 24 hours. Red lines (positioned at 3.0) indicate the minimum threshold to select significant markers (listed in S1 Table). The blue box in (c) highlights a portion of chromosome 8 with high concentration of significant associations. Arrows highlight major associations whose definition is given in Table 3. Marker positions were based on the reference genome of *M*. *truncatula Mt4*.*0v1* (SNPs not aligning were placed on a fictitious chromosome 9).

**Fig 2:**
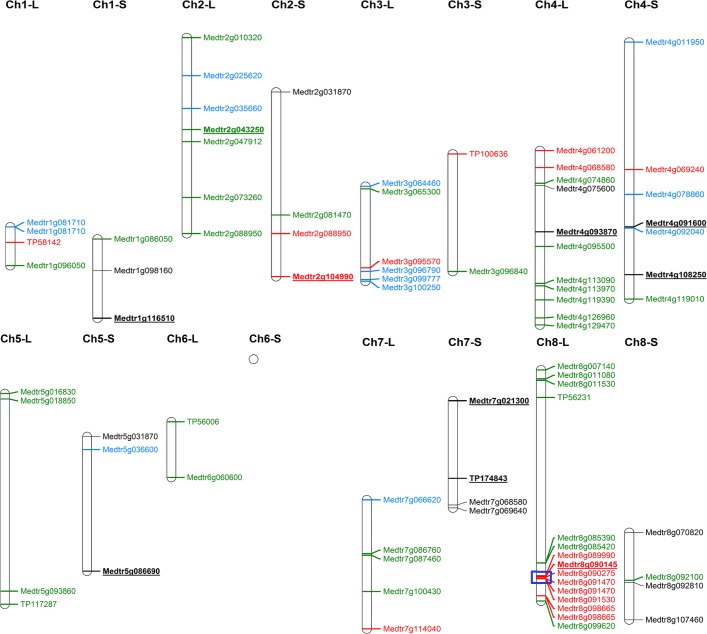
Physical map of annotated genes and markers associated with alfalfa leaf (L) and stem (S) quality traits. Blue, neutral detergent fiber; red, acid detergent lignin; black, crude protein; green, *in vitro* NDF digestibility at 24 hours. Major associations (whose definition is given in Table 3) are bold underlined. The blue box highlights a portion of chromosome 8 with high concentration of significant associations. Marker positions were based on the reference genome of *M*. *truncatula Mt4*.*0v1*.

The results of GWAS are summarized by Manhattan plots reported for each trait in Fig 1. Additional quantile-quantile plot results reported in S1 Fig indicated a manifest deviation from the expected *P*-value distribution only in the tail area, thereby suggesting that population stratification was adequately controlled. When fixing a threshold of 3.0 for significant association scores, the number of markers linked to a trait varied from a maximum of 42 (of which 30 mapped on *M*. *truncatula* genome *Mt4*.*0v1*) for leaf *in vitro* NDF digestibility at 24 hours, to a minimum of 2 (both mapped on *M*. *truncatula* genome *Mt4*.*0v1*) for leaf protein. In total, 83 significant SNPs were mapped on the reference genome, 53 of which for leaf traits, 26 for stem traits, one in common between organs, and three for leaf-to-stem ratio. Selected markers were assigned to all eight chromosomes. About 93% of these markers were located in or near (< 3000 bp) annotated genes (Fig 2, S1 Table).

Fig 2 reports the physical genome map with associations of annotated genes or markers with leaf or stem forage quality traits. Only three markers were associated with leaf-to-stem ratio, two of which located on chromosome 3 and one on chromosome 4 (data not shown). Two chromosomes (4 and 8) exhibited higher concentration of associations (Fig 2). In particular, a small genomic region (less than 900 Kbp) in chromosome 8 exhibited six SNP markers located in five annotated genes that associated with ADL in leaves (highlighted by blue boxes in Figs 1 and 2, and S1 Table).

Ten markers displaying high association score (-Log_10_(*P*-value) > 4.0) and coefficient of determination (*R*^2^ ≥ 0.10) are reported in Table 3 and highlighted by arrows in Fig 1. Six of them were associated with stem protein, whereas one major marker-trait association was found for leaf protein, leaf ADL, leaf NDFD and stem ADL (Table 3).

**Table 3:** Annotated genes or SNP markers associated with alfalfa forage quality traits with highest association score (-Log_10_(*P*-value) > 4.0) and coefficient of determination (*R*^2^ ≥ 0.10).

Trait^a^	Chromosome	SNP position (bp)	Gene context^b^	-Log_10_ (*P*-value)	*R*^2^	Gene	Annotation
L-ADL	8	37777925	C	4.18	0.115	Medtr8g090145	Serine/Threonine-kinase rio2
L-CP	4	37201603	C	4.43	0.114	Medtr4g093870	5-oxoprolinase
L-NDFD	2	18839740	C	5.17	0.100	Medtr2g043250	Auxin response factor
S-ADL	2	45249450	F	4.10	0.113	Medtr2g104990	NRAMP metal ion transporter 6
S-CP	1	52709070	C	4.13	0.108	Medtr1g116510	TMPIT-like protein
S-CP	4	36301957	C	4.81	0.128	Medtr4g091600	PPR containing plant-like protein
S-CP	4	44903265	C	4.80	0.128	Medtr4g108250	UDP-glucosyl-transferase protein
S-CP	5	37453146	C	4.56	0.121	Medtr5g086690	disease resistance protein, putative
S-CP	7	6734442	C	7.22	0.202	Medtr7g021300	disease resistance protein
S-CP	7	20442757	0	5.75	0.157	TP174843	

^a^L-ADL, leaf acid detergent lignin; L-CP, leaf crude protein; L-NDFD, leaf *in vitro* NDF digestibility at 24 hours; S-ADL, stem acid detergent lignin; S-CP, stem crude protein.

^b^C, coding sequence; F, 5’ and 3’ flanking regions (5’ and 3’ UTR included); 0, intergenic region.

In general, comparisons of results for same trait and chromosome suggested no co-localization of associations for the same trait in leaves and stems (Fig 2). In all cases but one (Medtr2g088950), markers were specifically associated with a single trait (i.e., they did not co-locate with other traits; Fig 2).

### Phenotype prediction for genomic selection

**Fig 3:**
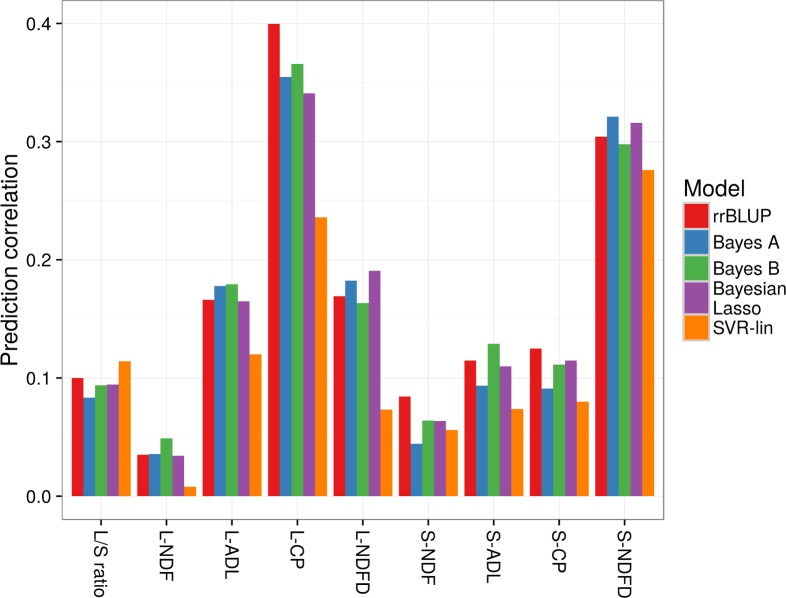
Accuracy of genome-enabled predictions for alfalfa forage quality traits based on five regression models. Pearson’s correlation between actual and predicted phenotypes (average of 500 repetitions of 10-fold cross validation). L/S ratio, leaf-to-stem ratio; L-NDF, leaf neutral detergent fiber; L-ADL, leaf acid detergent lignin; L-CP, leaf crude protein; L-NDFD, leaf *in vitro* NDF digestibility at 24 hours; S-NDF, stem neutral detergent fiber; S-ADL, stem acid detergent lignin; S-CP, stem crude protein; S-NDFD, stem *in vitro* NDF digestibility at 24 hours.

The average accuracy values (as correlation between actual and genome-predicted values) of five genomic selection models are reported in Fig 3 for each trait. In general, the models exhibited modest differences in accuracy for a given trait. However, the SVR-lin model displayed definitely lower accuracy for a few traits (leaf protein content and NDFD). Averaging accuracy values over traits indicated a trend for rrBLUP towards somewhat greater predicting ability (0.166), followed closely by Bayes B (0.161) and Bayesian Lasso (0.159).

Leaf protein content and stem NDFD displayed the best genome-enabled predictions, with accuracy values close to 0.4 and 0.3, respectively, for most-predictive models (Fig 3). Accuracy values were only modest for leaf ADL and NDFD, and low to very low for the other traits (Fig 3).

## Discussion

The three experimental growing conditions aimed to mimic some major alfalfa growing conditions in southern Europe and other Mediterranean-climate regions, where early-summer harvests may face drought depending on rainfed or irrigated cropping, whereas autumn harvests occur after the resumption of rainfall at the end of summer. Forage quality increased in autumn compared with summer harvests, as expected from higher leaf-to-stem ratio arising from earlier phenological stage of maturity in this season [63]. The absence of significant differences in forage quality traits between fully-irrigated and irrigation-suspended conditions was likely due to the limited drought stress produced by the latter condition (as indicated by the modest difference in mean forage yield between these conditions). The observed higher fiber and lignin content and lower protein content of stems relative to leaves is consistent with the known composition of the two tissues [64], and emphasizes the importance of stem quality improvement for alfalfa breeding.

Our reference genetic base likely represented the variation in forage quality traits that is commonly found in alfalfa breeding programs, as its component populations were chosen essentially on the basis of good forage yielding ability and adaptation to a given target region (here, the western Mediterranean basin; [21]) with no attempt to maximize the genetic variation for forage quality. Indeed, *CV*_*g*_ values never exceeded 7% for any trait and condition. For leaf-to-stem ratio, the current *CV*_*g*_ values (around 5–6%) are similar to those reported for half-sib progenies of a widely-based reference population from northern Italy [14] and local germplasm from Egypt [65]. Phenotyping half-sib progenies rather than cloned parent plants aimed to focus on the variation exploitable in breeding synthetic varieties, i.e., the additive genetic variation. Non-additive genetic effects, however, are reportedly modest for alfalfa quality traits [14, 66–68].

Information on the size of germplasm-by-environment interactions on alfalfa quality traits is scanty and controversial [69]. The current lack of interaction, and the high genetic correlation for progeny responses across conditions, suggests that selecting germplasm with good forage quality across different growing conditions is feasible.

The simultaneous performance of GWAS and genomic regression studies aimed to exploit different opportunities offered by these approaches. GWAS aimed to provide insight into the extent of polygenic control of each trait, as well as contributing to reveal major putative QTL and their association with annotated genes. Genomic regression maximizes the efficiency of selection based on many additive effects [57].

Interestingly, GWAS highlighted the lack of consistency between leaves and stems for SNP markers or annotated genes associated with a given trait. This finding, confirmed by low correlations between leaf and stem values, indicated substantially different genetic control of each quality trait in the two plant organs. GWAS identified six SNPs associated with stem protein content, of which five (spanning over four chromosomes) were located in the coding sequence of annotated genes. The importance of protein content and the weak concentration of proteins in the stems (e.g. 12.1% *vs* 26.5% in leaves across harvests [11]) encourages the exploitation of these SNPs for MAS, as well as the investigation of these potential candidate genes for stem protein content variation. In addition, GWAS highlighted a genomic area on chromosome 8 that was characterized by high concentration of SNP markers linked to ADL in leaves. Although lignin content is moderately low in leaves, improvement is favoured by the observed moderately large genetic coefficient of variation for this trait. A linked annotated gene, namely Medtr8g091470 (S1 Table), which is known to encode for a cellulose synthase interactive protein, was identified in that chromosome region by the presence of two SNPs in the same exon. This gene is similar (78% protein identity) to a cellulose synthase interactive protein in *Arabidopsis* (At2g22125) involved in fast recycling of cellulose synthase complexes [70]. This and other associated genes closely located on chromosome 8 (S1 Table) may deserve further study to verify their role as QTL for leaf lignin content control. The association with NDF content of an annotated gene observed recently for diploid germplasm [29] was not confirmed in our study.

On the whole, GWAS results confirmed the expected polygenic control of forage quality traits, thereby supporting the potential interest of genomic selection. Most of its models, particularly rrBLUP, Bayes B and Bayesian Lasso, displayed similar levels of accuracy for a given trait. We achieved moderate accuracy values for stem NDFD and leaf protein content (in the 0.3–0.4 range). The results for the former trait are of considerable practical interest, particularly for dairy systems, where NDFD is the main determinant of cattle dry-matter intake and milk yield [4]. Accuracy values for the other traits were probably too low for genome-enabled selection, at least in this reference population, whose genetic variation for quality trait was only moderate. Additional reasons for rather low accuracy levels could be the small size of the training population and the genetic complexity of the traits. For some traits, such as stem protein content and leaf or stem ADL, MAS based on a few associated markers highlighted by GWAS could be feasible. MAS or genomic selection may be poorly effective for a complex quality trait such as leaf-to-stem ratio and for NDF, according to our results.

In a previous study on the same germplasm set, genomic selection accuracy for forage dry-matter yield attained 0.35, implying definitely greater selection efficiency than field-based selection for forage yield breeding value in terms of genetic gain per unit time according to selection theory [21]. The current results add to previous ones, indicating that GBS data can be exploited to improve both quality traits (by genomic selection or MAS) and forage yield—thereby increasing the cost-effectiveness of marker-based selection procedures. The substantially different genetic control of a given trait in stems and leaves implied by some findings complicates forage quality improvement and reinforces the interest of improving the quality of stems, given their definitely lower intrinsic quality compared to leaves, their large weight proportion on alfalfa biomass, and the greater decrease of quality in stems than in leaves with maturity [3, 69].

## Supporting Information

S1 Fig Quantile-quantile plots resulting from GWAS for forage quality traits in alfalfa. Traits are: (a), leaf-to-stem ratio; (b), leaf neutral detergent fiber; (c), leaf acid detergent lignin; (d), leaf crude protein; (e) leaf *in vitro* NDF digestibility at 24 hours; (f), stem neutral detergent fiber; (g), stem acid detergent lignin; (h), stem crude protein; (i) stem *in vitro* NDF digestibility at 24 hours. The black line represents the expected values; the red line represents the λ adjustment for inflation.
(TIF)

S1 Table Physical localization of significant SNPs (association score > 3.0) and related annotated genes identified by GWAS. Blue-highlighted markers are those highlighted by blue boxes in Figs 1 and 2. ^a^L/S ratio, leaf-to-stem ratio; L-NDF, leaf neutral detergent fiber; L-ADL, leaf acid detergent lignin; L-CP, leaf crude protein; L-NDFD, leaf *in vitro* NDF digestibility at 24 hours; S-NDF, stem neutral detergent fiber; S-ADL, stem acid detergent lignin; S-CP, stem crude protein; S-NDFD, stem *in vitro* NDF digestibility at 24 hours. ^b^C, coding sequence; I, intron; F, 5’ and 3’ flanking regions (5’ and 3’ UTR included); 0, intergenic region. ^c^-Log_10_(*P*-value), association score; ^d^*R*^2^, coefficient of determination.
(PDF)
