# Supplementary figures and images for: Genome-Wide Association Mapping and Genomic Selection for Alfalfa (Medicago sativa) Forage Quality Traits
Source: PLoS One. 2017 Jan 9;12(1):e0169234. doi: 10.1371/journal.pone.0169234 (PMC5222375; doi:10.1371/journal.pone.0169234)

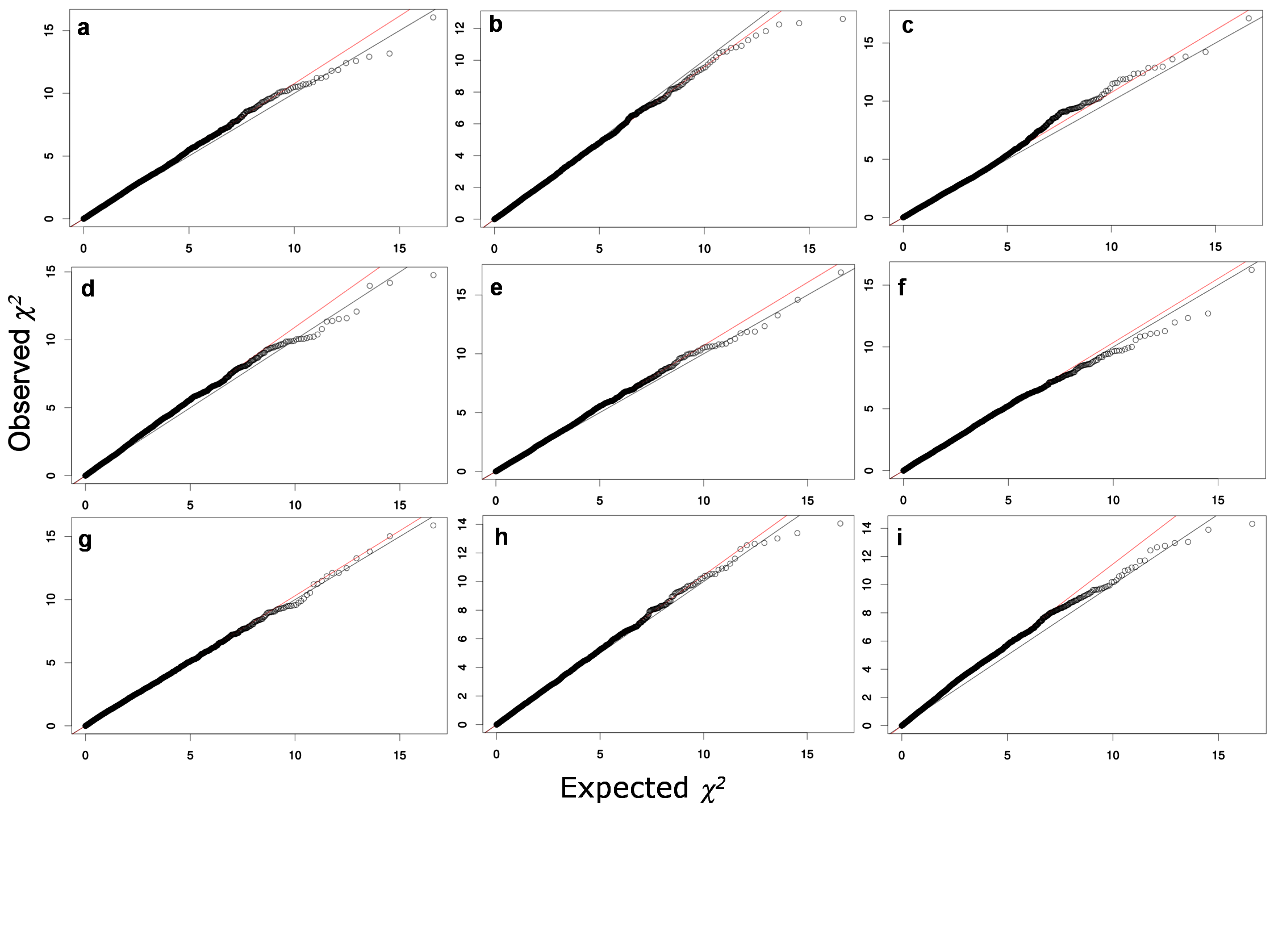

Supplement: S1 Fig — Traits are: (a), leaf-to-stem ratio; (b), leaf neutral detergent fiber; (c), leaf acid detergent lignin; (d), leaf crude protein; (e) leaf in vitro NDF digestibility at 24 hours; (f), stem neutral detergent fiber; (g), stem acid detergent lignin; (h), stem crude protein; (i) stem in vitro NDF digestibility at 24 hours. The black line represents the expected values; the red line represents the λ adjustment for inflation. (TIF) [file pone.0169234.s001.tif]
