# Supplementary material for: Genome-Wide Association Mapping and Genomic Selection for Alfalfa (Medicago sativa) Forage Quality Traits
Source: PLoS One. 2017 Jan 9;12(1):e0169234. doi: 10.1371/journal.pone.0169234 (PMC5222375; doi:10.1371/journal.pone.0169234)
Supplement: S1 Table — Blue-highlighted markers are those highlighted by blue boxes in Figs 1 and 2. aL/S ratio, leaf-to-stem ratio; L-NDF, leaf neutral detergent fiber; L-ADL, leaf acid detergent lignin; L-CP, leaf crude protein; L-NDFD, leaf in vitro NDF digestibility at 24 hours; S-NDF, stem neutral detergent fiber; S-ADL, stem acid detergent lignin; S-CP, stem crude protein; S-NDFD, stem in vitro NDF digestibility at 24 hours. bC, coding sequence; I, intron; F, 5’ and 3’ flanking regions (5’ and 3’ UTR included); 0, intergenic region. c-Log10(P-value), association score; dR2, coefficient of determination. (PDF) [file pone.0169234.s002.pdf]

S1 Table. Physical localization of significant SNPs (association score > 3.0) and related annotated genes identified by GWAS

Chromosome	SNP position (bp)	Trait[a]	Gene context[b]	$-Log_{10}$ (P-value)[c]	R^{2} [d]	Gene	Annotation
1	36328929	L-NDF	I	3.02	0.073	Medtr1g081710	armadillo/beta-catenin-like repeat protein
1	36328947	L-NDF	C	3.10	0.076	Medtr1g081710	armadillo/beta-catenin-like repeat protein
1	38493050	S-NDFD 24h	C	3.11	0.087	Medtr1g086050	preprotein translocase subunit SecA
1	39103325	L-ADL	0	3.27	0.086	TP58142	
1	43265277	L-NDFD 24h	C	3.00	0.029	Medtr1g096050	alpha/beta fold hydrolase
1	44174060	S-CP	C	3.43	0.087	Medtr1g098160	gigantea protein 1B s , regulation of circadian rhythm
1	52709070	S-CP	C	4.13	0.108	Medtr1g116510	TMPIT-like protein
2	2324687	L-NDFD 24h	F	3.91	0.059	Medtr2g010320	cytochrome P450 family protein
2	9163314	L-NDF	C	3.33	0.082	Medtr2g025620	transcription initiation factor IIF subunit alpha
2	12085565	S-CP	F	3.12	0.078	Medtr2g031870	myb transcription factor
2	15055656	L-NDF	C	3.00	0.073	Medtr2g035660	plastid transcriptionally active 14 protein
2	18839740	L-NDFD 24h	C	5.17	0.098	Medtr2g043250	Auxin response factor
2	21015186	L-NDFD 24h	C	3.05	0.020	Medtr2g047912	dentin sialophosphoprotein-like protein, putative
2	31031433	L-NDFD 24h	I	3.36	0.073	Medtr2g073260	trehalose-6-phosphate synthase
2	34191810	S-NDFD 24h	C	3.30	0.093	Medtr2g081470	S-locus lectin kinase family protein
2	37518080	S-ADL<br>L-NDFD 24h	C<br>C	3.30<br>3.33	0.088<br>0.034	Medtr2g088950	type II superfamily restriction endonuclease
2	45249450	S-ADL	F	4.10	0.113	Medtr2g104990	NRAMP metal ion transporter 6
3	23226535	S-ADL	0	3.63	0.099	TP100636	
3	29037685	L-NDF	F	3.23	0.079	Medtr3g064460	hypothetical protein
3	29478920	L-NDFD 24h	I	3.11	0.048	Medtr3g065300	chromosome condensation-like protein
3	43672555	L-ADL	I	3.56	0.095	Medtr3g095570	autophagy-related protein
3	44299319	L-NDF	C	3.00	0.072	Medtr3g096790	DnaJ domain protein- Auxilin-like protein
3	44327228	S-NDFD 24h	F	3.13	0.088	Medtr3g096840	transmembrane amino acid transporter family protein
3	45776379	L-NDF	C	3.17	0.078	Medtr3g099777	E3 ubiquitin-protein ligase UPL1-like protein
3	46072330	L-NDF	F	3.35	0.083	Medtr3g100250	hypothetical protein
3	51644704	L/S ratio	C	3.95	0.108	Medtr3g110460	transmembrane protein, putative

3	52357652	L/S ratio	C	3.33	0.088	Medtr3g111870	cytosolic purine 5-nucleotidase
4	3134901	S-NDF	C	3.21	0.083	Medtr4g011950	coatomer subunit beta-like protein
4	22616812	L-ADL	I	3.43	0.091	Medtr4g061200	AP2-like ethylene-responsive transcription factor
4	25662948	L-ADL	C	3.16	0.083	Medtr4g068580	phosphatidylinositol 3- and 4-kinase
4	26021055	S-ADL	F	3.54	0.096	Medtr4g069240	oligosaccharyltransferase 48 kDa subunit beta
4	28486551	L-NDFD 24h	I	5.78	0.069	Medtr4g074860	general transcription factor 3C-like protein
4	28889409	L-CP	I	3.16	0.077	Medtr4g075600	DNA-directed RNA polymerase II 8.2 kDa protein
4	30484111	S-NDF	C	3.32	0.086	Medtr4g078860	homeodomain transcriptional regulator
4	30698179	L/S ratio	C	3.04	0.080	Medtr4g079390	general transcription factor 3C-like protein
4	36301957	S-CP	C	4.81	0.128	Medtr4g091600	PPR containing plant-like protein
4	36527134	S-NDF	C	3.51	0.092	Medtr4g092040	FYVE zinc finger protein
4	37201603	L-CP	C	4.43	0.114	Medtr4g093870	5-oxoprolinase
4	39824533	L-NDFD 24h	C	3.77	0.046	Medtr4g095500	GRAS family transcription factor
4	44903265	S-CP	C	4.80	0.128	Medtr4g108250	UDP-glucosyltransferase family protein
4	46468556	L-NDFD 24h	I	3.60	0.071	Medtr4g113090	bile acid:sodium symporter
4	46898769	L-NDFD 24h	C	3.16	0.047	Medtr4g113970	zinc finger CCCH domain protein
4	49279375	S-NDFD 24h	C	3.39	0.096	Medtr4g119010	crooked neck protein/cell cycle protein, putative
4	49433130	L-NDFD 24h	I	3.17	0.068	Medtr4g119390	always EARLY-like protein
4	52656003	L-NDFD 24h	C	3.44	0.058	Medtr4g126960	membrane fusion protein Use1
4	53930083	L-NDFD 24h	C	4.04	0.053	Medtr4g129470	E3 ubiquitin-protein ligase XBOS32
5	6083584	L-NDFD 24h	C	3.66	0.010	Medtr5g016830	filament-like plant protein
5	7045149	L-NDFD 24h	C	3.02	0.025	Medtr5g018850	histone-lysine N-methyltransferase SUVR5-like protein
5	13669600	S-CP	C	3.15	0.079	Medtr5g031870	receptor-like cytosolic Serine/Threonine-kinase
5	15986057	S-NDF	C	3.00	0.075	Medtr5g036600	pyruvate dehydrogenase complex E1 alpha subunit
5	37453146	S-CP	C	4.56	0.121	Medtr5g086690	disease resistance protein (TIR-NBS-LRR class), putative
5	40982883	L-NDFD 24h	C	3.00	0.005	Medtr5g093860	microtubule associated protein, MAP65/ASE1 family protein
5	43301839	L-NDFD 24h	0	4.96	0.071	TP117287	
6	11070427	L-NDFD 24h	0	3.67	0.028	TP56006	
6	20902300	L-NDFD 24h	C	3.29	0.067	Medtr6g060600	ethylene insensitive 3 family protein
7	6734442	S-CP	C	7.22	0.202	Medtr7g021300	disease resistance response/dirigent-like protein
7	20442757	S-CP	0	5.75	0.157	TP174843	
7	24255620	L-NDF	C	3.41	0.085	Medtr7g066620	LRR receptor-like kinase
7	25135663	S-CP	F	3.15	0.079	Medtr7g068580	ubiquitin-protein ligase, putative

7	25671372	S-CP	C	3.94	0.102	Medtr7g069640	ammonium transporter 1 protein
7	33743562	L-NDFD 24h	F	3.64	0.061	Medtr7g086760	hypothetical protein
7	34041388	L-NDFD 24h	C	3.61	0.000	Medtr7g087460	global transcription factor
7	40438646	L-NDFD 24h	C	3.94	0.031	Medtr7g100430	ankyrin repeat plant-like protein
7	47033246	L-ADL	C	3.48	0.093	Medtr7g114040	histone H2A 6
8	1300294	L-NDFD 24h	C	3.16	0.048	Medtr8g007140	patatin-like phospholipase
8	2868717	L-NDFD 24h	C	3.23	0.038	Medtr8g011080	DnaJ heat shock amine-terminal domain protein
8	3131649	L-NDFD 24h	C	3.07	0.014	Medtr8g011530	asparagine-tRNA ligase
8	6162759	L-NDFD 24h	0	3.24	0.058	TP56231	
8	30002391	S-CP	C	3.01	0.075	Medtr8g070820	bZIP transcription factor family protein
8	35381591	L-NDFD 24h	F	3.93	0.028	Medtr8g085390	carbohydrate-binding X8 domain protein
8	35392375	L-NDFD 24h	C	3.01	0.114	Medtr8g085420	carbohydrate-binding X8 domain protein
8	37634495	L-ADL	I	3.17	0.083	Medtr8g089990	polyadenylate-binding protein II- Splicing factor RNPS1
8	37777925	L-ADL	C	4.18	0.115	Medtr8g090145	Serine/Threonine-kinase rio2
8	37925953	L-ADL	C	3.08	0.081	Medtr8g090275	MFS transmembrane transporter
8	38158696<br>38159932	L-ADL	C<br>C	3.05<br>3.16	0.080<br>0.083	Medtr8g091470	cellulose synthase-interactive protein
8	38200365	L-ADL	C	3.00	0.078	Medtr8g091530	SAP domain protein
8	38466402	S-NDFD 24h	C	3.00	0.083	Medtr8g092100	hypothetical protein
8	38817320	S-CP	C	3.44	0.087	Medtr8g092810	myb transcription factor
8	41187583<br>41187587	L-ADL	C<br>C	3.29<br>3.27	0.087<br>0.086	Medtr8g098665	anaphase-promoting complex subunit 11 RING-H2 finger protein
8	42122569	L-NDFD 24h	F	3.00	0.079	Medtr8g099620	1-acyl-sn-glycerol-3-phosphate acyltransferase, putative
8	45430552	S-CP	C	3.00	0.074	Medtr8g107460	tetratricopeptide domain thioredoxin

Blue-highlighted markers are those highlighted by blue boxes in Figs 1 and 2.

[a]L/S ratio, leaf-to-stem ratio; L-NDF, leaf neutral detergent fiber; L-ADL, leaf acid detergent lignin; L-CP, leaf crude protein; L-NDFD, leaf *in vitro* NDF digestibility at 24 hours; S-NDF, stem neutral detergent fiber; S-ADL, stem acid detergent lignin; S-CP, stem crude protein; S-NDFD, stem *in vitro* NDF digestibility at 24 hours.

[b]C, coding sequence; I, intron; F, 5’ and 3’ flanking regions (5’ and 3’ UTR included); 0, intergenic region.

[c]-Log_{10}(*P*-value), association score.

[d]R^2, coefficient of determination.
